# Omadacycline for management of *Mycobacterium* *abscessus* infections: a review of its effectiveness, place in therapy, and considerations for use

**DOI:** 10.1186/s12879-022-07857-7

**Published:** 2022-11-22

**Authors:** Ashley R. Rizzo, Nader H. Moniri

**Affiliations:** 1 Department of Pharmacy Practice, College of Pharmacy, Mercer University Health Sciences Center, Mercer University, Atlanta, GA 30341 USA; 2 Department of Pharmaceutical Sciences, College of Pharmacy, Mercer University Health Sciences Center, Mercer University, 3001 Mercer University Drive, Atlanta, GA 30341 USA; 3 Department of Biomedical Sciences, School of Medicine, Mercer University Health Sciences Center, Mercer University, Macon, GA 31207 USA; 4 Present Address: Department of Pharmacy, University of Washington Medical Center Montlake, 1959 NE Pacific St, Seattle, WA 98195 USA

**Keywords:** Omadacycline, Non-tuberculous mycobacteria, *Mycobacteriumabscessus*, *Mycobacterium abscessus* complex

## Abstract

The *Mycobacterium* *abscessus* complex (MABC) is a group of acid-fast, rapidly dividing non-tuberculous mycobacteria (NTM) that include a number of clinically important subspecies, including *M.* *abscessus,* *M.* *bolletii,* and *M.* *massiliense*. These organisms are prevalent in the environment and are primarily associated with human pulmonary or skin and skin structure infections (SSSI) but may cause more deep-seeded disseminated infections and bacteremia in the immunocompromised. Importantly, these NTM are resistant to most first-line anti-tuberculous agents and, due to intrinsic or acquired resistance, exhibit exceedingly low, variable, and geographically distinct susceptibilities to commonly used antibacterial agents including older tetracyclines, macrolides, aminoglycosides, cephalosporins, carbapenems, and sulfamethoxazole-trimethoprim. Omadacycline is a novel third-generation member of the tetracycline family of antibacterials that has recently been demonstrated to have potent anti-NTM effects and clinical efficacy against MABC, including *M.* *abscessus.* The purpose of this review is to present a comprehensive and up-to-date assessment on the body of literature on the role of omadacycline for *M.* *abscessus* infections. Specifically, the in vitro and in vivo microbiology, mechanisms of action, mechanisms of resistance, clinical pharmacokinetics, clinical efficacy, adverse effects, dosage and administration, and place in therapy of omadacycline in management of *M.* *abscessus* infections will be detailed.

## Background

*Mycobacterium* *abscessus* is an acid-fast, non-tuberculous mycobacteria (NTM) that is found ubiquitously in the environment and differs from most mycobacteria due to its rapid-growth characteristics [1–3]. Related subspecies including *M.* *massiliense* and *M.* *bolletii*, together with *M.* *abscessus,* make up the *Mycobacterium* *abscessus* complex (MABC). MABC can cause a variety of human diseases including pulmonary [3–7], skin and skin structure infections (SSSI) [8–11], central nervous system [12–15], ocular [16–21], and disseminated infections [22–25]. Disseminated disease most commonly affects patients that are immunocompromised or have underlying comorbidities [26]. In particular, patients with chronic pulmonary diseases such as cystic fibrosis (CF) are at significantly elevated risk for MABC-linked pulmonary infections [27–29], and the incidence of NTM infections in CF patients has increased nearly seven-fold since the turn of the century, contributing significantly to morbidity and mortality in CF patients [30, 31]. Moreover, those with non-CF chronic pulmonary diseases such chronic obstructive pulmonary disease (COPD) or bronchiectasis are also at higher risk for MABC infections [32–36].

Of the mycobacterium, which are historically more difficult to treat with chemotherapeutics than typical Gram-positive or Gram-negative organisms, *M.* *abscessus* is particularly challenging to treat due to its multi-drug resistant nature, as well as due to bacterium-specific characteristics. With regard to the former, *M.* *abscessus* are resistant to typical antimycobacterial agents such as rifamycins, isoniazid, pyrazinamide and ethambutol, and have extremely low, variable and geographically distinct susceptibilities to a variety of antibacterials including macrolides, aminoglycosides, cefoxitin, imipenem, sulfamethoxazole-trimethoprim (SMX-TMP), and tigecycline [37–39]. As discussed further below, extreme multi-drug resistance to these and other agents through intrinsic or acquired means has made treatment of *M.* *abscessus* extremely difficult. In addition to these drug resistance issues, treatment of *M.* *abscessus* has also faced significant obstacles due to bacteria-specific characteristics, including the organism’s ability to readily form biofilms, particularly within the human airway [40–42]. MABC biofilms allow for persistent bacterial colonization within a stable extracellular matrix in the airway, and have also been hypothesized to contribute to decreased immune cell function at these sites of colonization and infection [43, 44]. In CF patients, *M.* *abscessus* biofilms have also been shown to have variable mechanics, including different viscoelastic properties, that can alter colony morphology in the airway, and contribute to pathogenesis [45]. Together, biofilm formation has also been suggested to be linked directly to poor cure rates of MABC infections upon treatment with drugs with otherwise acceptable in vitro potency against the offending NTM [41, 42].

In addition, the prevalence of the MABC organisms in the environment, as well as their expiration from the airway and potential for person-to-person transmission either through airway droplets or from fomite contact adds to the potential dangers of MABC infection [2, 46]. For example, the presence of the organism in municipal drinking water sources, hospital water systems, and even industrial cooling towers [2, 47–50] increases the likelihood of exposure and colonization, even in non-developing nations with appropriately hygienic water supply infrastructure. Moreover, soil and dust-derived exposures can increase the risk of environmentally-sourced infections [51–53], as can the organism’s fitness towards causing transmission through fomite contact [54], and together, these obstacles make prevention and treatment of MABC infections a formidable challenge. Given these issues, development of novel agents that circumvent MABC resistance mechanisms is an unmet and highly warranted need. Omadacycline (Nuzyra®), a newer third-generation tetracycline, has been recently shown to exhibit effective clinical utility against *M.* *abscessus*, and other MABC members. Omadacycline is a first-in-class aminomethylcycline, structurally related to the tetracycline family of antimicrobials that includes tetracycline, minocycline, doxycycline and the newer glycylcycline member tigecycline [55]. The purpose of this review is to provide a comprehensive appraisal on the use of omadacycline for treatment of *M.* *abscessus*, including its pharmacology, pharmacokinetics, clinical efficacy, and adverse effects.

## Main text

### Microbiological spectrum

Omadacycline activity against a variety of *M.* *abscessus* isolates were consistently equipotent or more potent compared to tigecycline, with in vitro Minimal Inhibitory Concentration (MIC_50_) of 1 μg/ml against 16 isolates [56]. Along similar lines, Shoen and colleagues demonstrated in vitro MIC_50_ values of 0.25–2 μg/ml against 24 M*.* *abscessus* isolates [57] (Table 1). In seven day exposure in vitro assays, omadacycline demonstrated concentration-dependent anti-*M.* *abscessus* activity with bacteriostatic effects exerted at 4 μg/ml and bactericidal effects at concentrations ≥ 16 μg/ml, and no evidence of inducible resistance [58]. Concentration and time-dependent killing during this time frame has been reported against twelve *M.* *abscessus* isolates by others as well [59]. Interestingly, a recent study demonstrates significantly higher *M.* *abscessus* MIC_50_’s of 0.12 μg/ml against twenty isolates, and the authors suggest that assay-specific differences (shorter three day treatments performed at lower temperatures) or geographic differences between isolates could explain the nearly ten-fold differences [60]. This high degree of potency was also illustrated in twelve clinical *M.* *abscessus* isolates that revealed a median omadacycline MIC of 0.25 μg/ml, and which decreased to 0.5 μg/ml, depending on the culture broth used [59], further substantiating apparent differences in potency based on growth conditions. Importantly, omadacycline demonstrated potency against strains that displayed high-level resistance to a number of antibacterials used to treat *M.* *abscessus* infections, including clarithromycin, azithromycin, amikacin, cefoxitin, imipenem, and also synergized the effects of clarithromycin, azithromycin, cefdinir, and linezolid against several isolates [59]. The potentiation effect with clarithromycin has also been recently demonstrated by others and revealed synergistic effects on MIC of both omadacycline and clarithromycin in the presence of each other compared to either agent alone [61]. In a murine model of pulmonary *M.* *abscessus* infection, the reference ATCC19977 strain, which is resistant to linezolid and moxifloxacin, and three isolates representing distinct clinical susceptibility phenotypes that also include resistance to amikacin and imipenem, were used to assess omadacycline efficacy [59]. Results showed that after 4 weeks of treatment, omadacycline reduced *M.* *abscessus* lung burden by approximately 1–3 log units with no change in MICs over the course of treatment, suggesting high efficacy of omadacycline for treatment of *M.* *abscessus* pulmonary infections [59]. Omadacycline was also noted to demonstrate potent in vitro effects against 22 isolates of *Mycobacterium* *chelonae* and 20 isolates of *M.* *fortuitum* *spp.*, with MIC_90_ of 0.250 μg/ml and 0.5 μg/ml, respectively [57] (Table 1). These values were similar to that seen with tigecycline, but 32- and 256-fold more potent than amikacin and doxycycline, respectively [57].

**Table 1:** In vitro potency and performance standards for susceptibility testing

Organism	MIC ranges (μg/ml)	
	Omadacycline	Tigecycline
*Mycobacterium* abscessus^*^	0.06–2	0.06–2
*Mycobacterium* chelonae^*^	0.015–0.25	0.03–0.125
*Mycobacterium* fortuitum^*^	0.03–1	0.03–1
*Escherichia* *coli*^**†**^	0.25–2	0.03–0.25
*Staphylococcus* *aureus*^**†**^	0.12–1	0.03–0.25
*Enterococcus* *faecalis*^**†**^	0.06–0.5	0.03–0.12
*Bacteroides* *fragilis*^**†**^	0.12–1	0.06–0.5
*Clostridioides* *difficile*^**†**^	0.06–0.25	0.03–0.12

^*^Data from Shoen et al. [57]

^**†**^Data from CLSI [97]

In addition to Mycobacterium species, the in vitro antimicrobial effects of omadacycline include a broad spectrum of coverage against other difficult to treat organisms, including methicillin-resistant *Staphylococcus* *aureus* (MRSA), vancomycin-resistant enterococci (VRE), extended-spectrum β-lactamase (ESBL)-producing *Enterobacteriaceae*, carbapenem-resistant *Enterobacteriaceae* (CRE), and multidrug-resistant *Acinetobacter* species [62–64]. Omadacycline was reported to have potent (≤ 1 μg/mL) in vitro activity against > 99% of isolates of gram-positive aerobes, including Methicillin-sensitive *Staphylococcus aureus * (MSSA), Methicillin-resistant *Staphylococcus aureus* (MRSA), Vancomycin-resistant *Enterococcus* (VRE), including vancomycin-resistant *E.* *faecium* and *E.* *faecalis*, and even greater potency (≤ 250 ng/mL) against 99% of isolates of *S.* *pneumoniae* *and* *S.* *viridans,* including penicillin and tetracycline resistant species, as well as *S.* *agalactiae*, including erythromycin-resistant strains [62]. Against gram-negative aerobes, omadacycline exhibits lower potency, with MICs of 1–4 μg/mL against *E.* *coli,* *K.* *pneumoniae,* *E.* *cloacae,* *Citrobacter* *spp.,* *P.* *aeruginosa,* *H.* *influenzae,* *and* *P.* *mirabilis*, including respective ESBL and CRE Enterobacteriaceae phenotypes, as well as ceftazidime resistant strains [64]. Similar to its potent effects against Mycobacterium, omadacycline exhibits potent in vitro activity against non-mycobacterial atypical organisms, including *Mycoplasma* *pneumoniae* and *hominis*, *Legionella* *pneumophilia*, and *Chlamydia* *pneumoniae*, with MIC_90_s in the range of 0.06–0.25 μg/ml, which are two-fold to over 30-fold greater than that seen with doxycycline for these organisms [57, 63–66]. Omadacycline also displays moderate in vitro activity against anaerobes, including *Bacteroides* *spp.*, where MIC_90_s ranged from 1–4 μg/mL, while it exhibited much more potent effects against *Clostridioides* *difficile,* with MIC_90_ of 0.5 μg/mL [67].

### Pharmacology and resistance

Omadacycline is the first-in-class aminomethylcycline, structurally related to the tetracycline family of antimicrobials that includes tetracycline, minocycline, doxycycline and the newer glycylcycline member tigecycline. Consistent with this, omadacycline exerts its antibacterial effects via binding to the tetracycline-binding site of the bacterial 16S ribosomal RNA, inhibiting bacterial protein synthesis with an in vitro potency similar to that of minocycline [68]. Clinically, distinction of *M.* *abscessus* from other mycobacterium or subspecies within the MABC is important due to differences in resistance, particularly inducible resistance. For example, *M.* *abscessus* expresses a unique *erm*(41) gene that confers resistance to macrolides due to ribosomal methylation, and which is not expressed or is non-functional in related MABC subspecies [69, 70]. Importantly, exposure to the macrolide-lincosamide-streptogramin-ketolide agents including clarithromycin, clindamycin, quinupristin, and telithromycin, respectively, yielded 23–250-fold upregulation of *erm*(41) expression in 24 h, demonstrating robust inducible resistance to these agents in *M.* *abscessus* [69]. Interestingly, agents within the same class may confer higher levels of resistance to other agents within that class, as seen with clarithromycin and azithromycin [71]. Acquired resistance to macrolides can also occur due to point mutations in the *rrl* gene, which encodes for amino acids that make up the peptidyltransferase site of the target 23S ribosomal RNA [70]. Similarly, high level resistance to aminoglycosides including amikacin and tobramycin can occur due to rapid single-point mutation of the *M.* *abscessus* 16S ribosomal RNA, encoded for by the *rpsL* gene [72, 73], as well as the more conventional mechanism that occurs via modification of aminoglycosides by plasmid-conferred acetyltransferase or phosphotransferase enzymes expressed by *M.* *abscessus* [74–76]. With regard to cefoxitin and imipenem, mutations of the target PBP genes and expression of extended-spectrum β-lactamases can confer acquired resistance to mycobacterium, while lack of penetration to the cell wall or the ability of *M.* *abscessus* to utilize *D,D*-transpeptidases that are not efficiently recognized by β-lactam antibacterials can facilitate intrinsic resistance in some isolates [77–80].

Mycobacteria, including *M.* *abscessus,* have also acquired resistance to tetracyclines primarily via expression of ribosomal protection proteins or efflux transporters. Expression of the *otr(*A), *tet*(M), *tet*(O) gene products confers protection to the ribosomes by facilitating translation in the presence of the tetracycline, while a variety of efflux transporters have also been identified that can promote intracellular tetracycline removal [81]. Interestingly, current evidence suggests that ribosomal protection and efflux mechanisms are not prevalent towards resistance to the third-generation glycylcycline tigecycline, another relatively newer member of the tetracycline family, which can overcome resistance to ribosomal protection and efflux. Consistent with this, tigecycline displays good in vitro activity against *M.* *abscessus*, and this effect translates to a significant degree of clinical susceptibility of *M.* *abscessus* isolates to this agent [37, 73, 82–84]. In a similar manner, omadacycline exhibits potent antibacterial effects against bacterial strains which express *tet*(K) efflux transporters, as well as *otr*(A), *tet*(M) and *tet*(O) ribosomal protection proteins [81], and with 17–180-fold greater potency than tetracycline against these resistant strains [68].

### Clinical disposition and pharmacokinetics

#### Plasma concentrations

In phase I studies conducted in healthy volunteers, a single 300 mg oral (PO) dose of omadacycline led to a plasma C_max_ of 563 ± 79.5 ng/mL and AUC_last_ of 8573 ± 1941 ng∙h/mL [85] and these were in line with other studies, including phase III trials (548 ± 146 ng/mL and 9399 ± 2559 ng∙h/mL, respectively) [86, 87]. At steady state, 300 mg PO dosing resulted in a C_max_ of 952 ± 420 ng/mL and AUC_last_ of 11,156 ± 5010 ng∙h/mL, with an accumulation ratio of 1.5, indicating significant accumulation [86]. A 450 mg PO dose displayed equivalently higher C_max_ of 1077 ± 269 ng/mL and AUC of 3367 ± 3469 ng∙h/mL at steady state. Intravenous (IV) dosing with 100 mg omadacycline exhibited similar single-dose and steady state AUC values as the 300 mg dose, while the C_max_ of 1507 ± 582 and 2116 ± 680, respectively, was expectedly higher compared to the PO route of administration [86]. Phase III trial data also revealed a T_max_ of 0.6, 2.5, and 2.5 h for the respective single-dose 100 mg IV, 300 mg PO, and 450 mg PO regimens, which were indifferent at steady state [86–88].

#### Oral absorption

Omadacycline exhibits poor (34.5%) bioavailability upon PO administration [85, 87] and this effect is significantly further decreased upon feeding, which essentially requires PO administration in the fasted state for proper systemic absorption. Omadacycline bioavailability is reduced by ~ 40% by a non-dairy high fat meal eaten 2 h prior to PO administration and by ~ 15% if eaten four hours prior [89], hence, a longer comparative fast than the typical “one hour before or two hours after a meal” is recommended. As expected based on chelation of tetracyclines by cations, including Ca^+2^, consumption of a dairy-containing meal within 2 h of administration reduced PO bioavailability by ~ 60%, versus the fasted-state [89], suggesting that dairy and other dietary chelators should be spaced by at least 4 h.

#### Distribution

Omadacycline is widely distributed and preclinical studies showed that concentrations in most tissues, including the skin, lymph nodes, liver, lungs, and kidneys after a single IV or PO dose exceed those in the blood within 24 h [90]. Unsurprisingly and as expected based on the affinity of tetracyclines for Ca^+2^, bone mineral contained the highest concentration of a single dose, over 130-fold that in the blood [90]. At steady state, omadacycline exhibited higher distribution into pulmonary epithelial lining fluid and lung alveolar cells compared to tigecycline in healthy adult studies, supporting its effectiveness in pneumonia [91]. Unlike other members of the tetracycline family that demonstrate higher levels of plasma protein binding [92], omadacycline only weakly binds human plasma proteins and the total protein bound fraction of 20% is not concentration-dependent [90].

#### Metabolism and elimination

Omadacycline is not metabolized or biotransformed to a significant degree in humans and analysis of a single [^14^C]-labeled dose showed the major fraction is unchanged omadacycline, while the product of 4-dimethylamine epimerization, typical of all tetracycline members, was the only other identified major product, but was also identified in the drug substance with other trace impurities [85]. Of the radioactive PO dose, ~ 81% was excreted in the feces, and this was mainly attributed to unabsorbed drug. Meanwhile, 14% of the PO dose was excreted in the urine, representing approximately 40% of the absorbed dose [85]. Over 95% of radioactivity was recovered within 168 h, and the 92% of the fecal excretion occurred within 72 h [85]. Omadacycline was not found to be a substrate, inhibitor, or inducer of the major CYP enzymes, consistent with other members of the tetracycline class, however, the lack of metabolic transformation is unique, particular given the biotransformation of tigecycline by phase I and phase II metabolism [93]. The elimination half-life of omadacycline after a single PO dose of 300 mg or 450 mg, or a single IV dose of 100 mg is in 13–16 h range [86, 87, 94]. Renal clearance of omadacycline (mean 3.1 L/h) is unaffected by renal function, demonstrating that no dosing adjustment is required for patients with renal dysfunction or during hemodialysis [95]. Similar results were found in patients with mild or moderate hepatic impairment [96].

### Breakpoints

Similar to tigecycline, to date, no definitive susceptibility test interpretive criteria breakpoints, as defined by Clinical and Laboratory Standards Institute, have been established for omadacycline against *M.* *abscessus* [43]. For other susceptible organisms, omadacycline susceptibility breakpoints are similar to, or are slightly less potent than tigecycline (Table 1) [97]. For tigecycline, isolates of *M.* *abscessus* are susceptible to concentrations less than or equal to 2 μg/ml [98–100], leading to proposed tigecycline susceptibility breakpoints of 0.5 to 4 μg/ml [101, 102]. Omadacycline MICs were demonstrated to be at least equal to, and in some cases, twofold lower than those of tigecycline against ten of 14 MABC isolates [57], suggesting similar breakpoints to those proposed for tigecycline until formal recommendations are put in place.

### Clinical efficacy

To date, two case reports and two case series have been published in the literature to assess the real-world utilization of omadacycline for treatment of NTM infections, specifically those involving *M.* *abscessus,* *M.* *chelonae,* *M.* *Bolleti,* *and* *M.* *massiliense,* and these results are summarized in Table 2. Minhas and colleagues published a case report assessing utilization of omadacycline in a resistant pulmonary *M.* *abscessus* infection of a 67-year-old Chinese female with documented drug allergies to several antimicrobials, including imipenem, tigecycline, and others [103]. Additionally, the patient failed previous treatment for chronic bronchitis from *Mycobacterium* *abscessus* with aztreonam monotherapy and later in combination with doxycycline. The patient was initiated on omadacycline 150 mg PO daily, amikacin 500 mg IV three times weekly, and aztreonam 1000 mg IV every 8 h for 4 weeks, and a follow-up visit 4 weeks later revealed that this regimen had been well tolerated with no report of adverse events [103]. She demonstrated improvement in cough and shortness of breath and chest imaging showed stable disease without progression, and the authors concluded that omadacycline could be utilized as part of a multi-drug regimen for treatment of NTM infections.

**Table 2:** Published clinical studies on omadacycline (OMA) use for *Mycobacterium abscessus* complex

Study and OMA treatment regimen (*n*)	NTM Species (*n*)	Infection source (*n*)	Duration of OMA therapy
Minhas et al. [103] OMA 150 mg PO daily	*M.* *abscessus*	Pulmonary	4 weeks
Frizzell et al. [104] OMA 450 mg PO on days 1-2 then 300 mg PO daily	*M.* *chelonae*	Cutaneous	4 months
Pearson et al. [105] Case 1: OMA 450 mg PO on days 1-2 then 300 mg PO daily	Case 1: *M.* *bolletii*	Case 1: Cutaneous	Case 1: 6 months
Case 2: OMA 450 mg PO on days 1-2 then 300 mg PO daily	Case 2: *M.* *abscessus*	Case 2: Pulmonary	Case 2: 7 months
Case 3: OMA 300 mg PO daily	Case 3: *M.* *abscessus*	Case 3: Cutaneous	Case 3: 6 months
Case 4: OMA 300 mg PO daily	Case 4: *M.* *abscessus*	Case 4: Blood/bone	Case 4: 3 months
Morrisette et al. [106] OMA 450 mg PO on days 1-2 then 300 mg PO daily (2) OMA 300 mg PO daily (10)	M. abscessus complex; no subspecies (5) *M.* *abscessus* (6) *M.* *massiliense* (1)	Pulmonary (7) Bone (2) Intra-abdominal (1) Cutaneous (1) Catheter (1)	IQR 4.2–11.0 months

Frizzell and colleagues published a recent case report on utilization of omadacycline in treatment of a *Mycobacterium* *chelonae* (*M.* *chelonae*) skin infection in a paraplegic 52-year-old female with an extensive history of non-healing lower extremity ulcers and new cutaneous nodules [104]. This species of NTM can cause pulmonary and cutaneous disease and is often multi-drug resistant, similar to other rapid growing NTM, such as *Mycobacterium* *abscessus*. Cultures of fluid obtained from these wounds were positive for *M.* *chelonae* with resistance to several agents including clarithromycin. Omadacycline MIC was reported at 0.25 μg/mL and omadacycline was initiated to avoid hospitalization for IV agents. A loading dose of 450 mg PO on day one and day two was utilized, followed by 300 mg PO daily thereafter for a total of 4 months. Upon follow-up in clinic 4 weeks later, dramatic improvement of lesions was noted without any report of adverse events or need for discontinuation of therapy [104]. The authors concluded that omadacycline was efficacious against *M.* *chelonae* SSSI.

A retrospective case series review on utilization of omadacycline for treatment of MABC disease in four patients was also recently published [105]. Patients were included if they received omadacycline for treatment of culture positive *M.* *abscessus* and susceptibilities were completed for all cases. Of the cases, two were cutaneous infection, one was pulmonary infection, and one was bacteremia with osteomyelitis. All patients underwent surgical intervention in combination with antimicrobial therapy and all patients received at least 3 months of omadacycline. Although omadacycline was utilized for less than half of total treatment duration, and was generally added later in treatment due to progression of disease or adverse effects to prior agents, it did result in clinical resolution in three of the four patients, with the fourth improving upon ongoing treatment, and overall favorable tolerability over 7 months [105]. A summary of each case is provided here:

Patient One had cutaneous MABC (subspecies *M.* *bolletii*) after receiving breast implants and was treated with various antimicrobials, including clarithromycin/SMX-TMP, and later clarithromycin/linezolid, with disease progression continuing in both. Due to a reported MIC of 0.25 μg/mL to tigecycline, susceptibility was inferred to omadacycline and the patient received omadacycline 450 mg PO daily for two days, followed by 300 mg PO daily in combination with tedizolid and azithromycin for 6 months without report of adverse events. She had no evidence of recurrent infection 8 months after completion of therapy.

Patient Two had pulmonary MABC (subspecies *M.* *abscessus*) and received initial induction treatment with amikacin, imipenem, clofazimine, and omadacycline 450 mg PO for 2 days, then 300 mg PO daily thereafter. He underwent lobe resection 5 months into therapy due to continued symptoms of disease, but biopsy was free of acid-fast bacilli so therapy was concluded 3 months after resection. The patient tolerated omadacycline therapy for over 7 months without report of adverse events. He had no evidence of recurrent infection 4 months since completion.

Patient Three had a cutaneous polymicrobial infection with MABC (subspecies *M.* *abscessus*) after liposuction procedure, found to be resistant to linezolid and macrolides. Omadacycline 300 mg PO daily without a loading dose was utilized in combination with azithromycin and clofazimine. Repeat operative cultures were negative, however, omadacycline was discontinued 6 months into therapy due to nausea and vomiting.

Patient Four, who presented with a complex history including Guillain-Barré syndrome, erythromelalgia, prior chronic antibiotic therapy for Lyme disease, and below-the-knee amputation due to chronic inflammatory demyelinating polyneuropathy, was found to have MABC (subspecies *M.* *abscessus*) positive blood cultures in addition to positive bone and tissue cultures suggestive of discitis and osteomyelitis. The treatment regimen was adjusted several times due to ongoing discitis and development of abscess, however, fluid aspiration cultures were negative at 60 days. The final treatment regimen included omadacycline 300 mg PO daily without loading dose and bedaquiline for a total of two years of therapy; of this, 15 weeks of treatment included the use of omadacycline without reports of adverse events. There was no evidence of infection recurrence 6 months since therapy completion.

Recently, Morrisette and colleagues published a multicenter, retrospective, observational case series of twelve adults who received omadacycline for at least 3 months from January through August of 2020 and had positive pulmonary or extrapulmonary cultures for MABC [106]. The primary outcome was a composite of survival, lack of clinical or radiological worsening, lack of omadacycline therapy alteration due to treatment failure, lack of microbiological relapse, and lack of culture positive persistence for three or more consecutive cultures. Most patients were a median age of 58 years, Caucasian (92%), primarily had pulmonary NTM sources (58%), and most isolates were subspecies *M.* *abscessus* (86%) expressing the *erm*(41) gene (66%), which had undergone tigecycline susceptibility testing (92%). Median duration of omadacycline therapy was 6.2 months with median follow-up duration of 5.1 months, with all participants receiving two or more antimicrobials in combination with the most common being amikacin (67%). Only PO omadacycline was utilized and only two of twelve patients received a 450 mg loading dose on days one and two with all participants receiving maintenance doses of 300 mg daily. Clinical success was achieved in nine cases, with failures documented in two pulmonary cases and one skin and skin structure infection. Adverse effects related to omadacycline were reported in three participants; gastrointestinal upset which was alleviated with dose adjustment to 150 mg twice daily, a temporary discontinuation due to elevated serum creatinine, and elevated liver function tests which were self-limiting and did not require adjustment or discontinuation of therapy.

### Adverse events and drug interactions

Utilization of omadacycline as part of combination therapy for *M.* *abscessus* infections has been highlighted for the lower incidence of, and a generally less severe, adverse effect profile compared to other recommended agents, although some relevant adverse effects have been noted in recent literature and within the package insert. Adverse events reported from recent literature with real-world utilization of omadacycline include nausea and vomiting [105], gastrointestinal upset, and elevation of serum creatinine [106]. Within the package insert, the most common (> 2%) adverse events reported from clinical trials include nausea, vomiting, infusion site reactions, alanine aminotransferase elevation, aspartate aminotransferase elevation, γ-glutamyl transferase elevation, hypertension, headache, diarrhea, insomnia, and constipation [86]. Several warnings and precautions have been highlighted in the package insert as a result of data from early clinical trials. In one study of patients with community-acquired bacterial pneumonia, the use of omadacycline was associated with a 2% mortality rate compared to 1% mortality rate with use of moxifloxacin [86]. The reasoning behind this imbalance was never identified so caution is advised when utilizing omadacycline for community-acquired pneumonia [86]. Similarly to other agents within the tetracycline class, warnings related to tooth discoloration, enamel hypoplasia, and inhibition of bone growth are considerations when omadacycline is used in pregnancy and early childhood [86]. Tetracyclines, including omadacycline, are subject to decreased absorption when co-administered with multivalent cation-containing preparations and have also been shown to depress plasma prothrombin activity which may require decreases in anticoagulant doses when used concomitantly [86]. Any known hypersensitivity to omadacycline itself or to other agents within the tetracycline class would be considered a contraindication to receiving omadacycline.

### Dosage and administration

Omadacycline is currently approved by the Food and Drug Administration (FDA) for treatment of community-acquired pneumonia and SSSI as both IV and PO formulations. IV administration of omadacycline is recommended at 200 mg as a single dose or 100 mg twice daily on day one, followed by 100 mg once daily thereafter [86]. PO administration of omadacycline varies based on infection type. For pulmonary infections, PO omadacycline is recommended as 300 mg twice daily on day one and 300 mg once daily thereafter. For SSSI, PO omadacycline is recommended as 450 mg once daily on days one and two, followed by 300 mg once daily thereafter. No dose adjustments are required in altered renal function or hepatic impairment [86].

Intravenous administration of omadacycline requires the infusion solution to be at room temperature prior to administration. Once reconstituted, diluted infusion solutions are stable for 24 h at room temperature and for seven days with refrigeration. All 200 mg doses should be infused over 60 min and 100 mg doses over 30 min through a dedicated Y-site; however, if no dedicated Y-site is available, the line should be flushed with normal saline or dextrose 5% before and after infusion of omadacycline [86].

PO administration of omadacycline should be done on an empty stomach after fasting for four or more hours and food or drink should be avoided for two hours after administration due to decreased absorption when administered with food. As with other tetracycline class members, dairy products or other products with multivalent cations should be avoided for four hours after administration to avoid chelation of drug [86].

### Place in therapy

Guidance on diagnosis, treatment, and prevention of NTM diseases comes from the American Thoracic Society (ATS) and the Infectious Diseases Society of America (IDSA) summary statement from 2007, the British Thoracic Society (BTS) guidelines from 2017, as well as review articles highlighting the role of newer agents in light of resistance and new drug development [37, 107, 108]. *M.* *abscessus* infections primarily manifest as cutaneous, bone, and pulmonary diseases, so treatment recommendations vary based on the specific infection site. Additionally, the consistent resistance patterns of *M.* *abscessus* to standard anti-tuberculous agents drives the recommendation for management of all these infections after susceptibility testing is completed for clinically significant isolates.

Serious skin and soft tissue and bone infections are recommended to be managed by the ATS/IDSA using clarithromycin or azithromycin, plus an initial parenteral agent for a minimum of 2 weeks (amikacin, imipenem, or cefoxitin) as part of macrolide therapy, pending clinical improvement [109]. In general, skin and soft tissue infections are recommended to be treated for a minimum of 4 months to provide high likelihood of cure. Bone infections are typically recommended to be treated for a 6-month duration [109].

Pulmonary disease recommendations include utilization of combination therapy as outlined above, with key differences in recommendations between guidelines. In contrast to the ATS/IDSA, the BTS recommends initial therapy with amikacin and tigecycline, plus imipenem and a macrolide, if tolerated, for a minimum of 1 month [110]. Furthermore, continued antimicrobial use after completion of the initial phase is recommended, including nebulized amikacin as well as 1–3 additional agents based on the susceptibility of the isolate, such as clofazimine, linezolid, minocycline, or others [110]. Although eradication and cure of *M.* *abscessus* via 12-month culture negative criteria in pulmonary disease is much less likely. In these instances, *M.* *abscessus* is managed for most patients as a chronic infection with intermittent periods of antimicrobial therapy and alternative goals including symptomatic improvement, regression of pulmonary infiltrates, or short periods of conversion to sputum culture negativity [109]. Recommended overall duration of treatment is for 12 months after culture conversion and some patients may be on long-term suppressive therapy do to failure to convert to negative cultures [110]. Due to the potential need for extended durations of parenteral agents with significant toxicities or in the setting of drug allergies, the role of omadacycline to allow for utilization of more PO agents or as an alternative to a more toxic agent could provide more flexibility in management of these chronic infections.

In addition to increasing the availability of active and tolerable agents used for treatment, utilization of PO omadacycline may help overcome barriers to discharge and medication access concerns encountered with parenteral antimicrobials. During selection of antimicrobial therapy for outpatient parenteral use, additional assessments must be made outside of only therapeutic considerations. For instance, patients with insurance coverage may be impacted by level of coverage provided for specific agents, out of pocket cost to the patient, or whether they must receive these agents in an infusion center or can self-administer at home [111]. As noted below, patients without insurance coverage may be limited by overall cost of drug therapy, obtaining and paying for home health services, or general access to parenteral agents.

Logistical and cost considerations notwithstanding, several safety concerns have been identified with parenteral administration of antimicrobials. Although the types of adverse events associated with parenteral agents does not differ from inpatient to outpatient, the overall incidence of these events increases with longer durations of therapy [111]. With courses of therapy for pulmonary *M.* *abscessus* infection lasting at least 12 months, the incidence of adverse events would be expected to be higher for these patients than in shorter courses of therapy. Finally, some specific agents utilized, such as amikacin, are subject to plasma concentration monitoring requirements to ensure they are within goal therapeutic and nontoxic ranges.

### Economic burden

Regardless of whether patients have prescription insurance coverage, the cost and availability of omadacycline may also be an important consideration towards its use. As a branded novel agent, the cost of Nuzyra is high compared to older, mainly generic, agents within the tetracycline class, and as a consequence, the agent must be obtained through the specialty pharmacy network of the manufacturer. Provided it is an available agent on their insurance formulary, patients can consider the use of the manufacturer’s patient assistance program, accessible through the Nuzyra Central web portal, that allows for copay-based cost reduction for commercially insured patients. In the instance patients do not have prescription coverage, the manufacturer also offers a patient support program to eligible individuals that may provide reimbursement and resources in covering the cost of this medication. These manufacturer resources may be beneficial for most patients who require this medication, as the average wholesale cost is in the range of $250–280 per 150 mg tablet [112].

## Conclusion

The global occurrence and associated morbidity of non-tuberculous infections caused by *M.* *abscessus* is increasing, and the organism has acquired or is intrinsically resistant to a variety of common antibacterials. Omadacycline covers a broad spectrum of clinically important organisms, including those within the MABC, where it has potent antimicrobial effects, particularly against *M.* *abscessus*. Since resolution of MABC infections often requires persistent chronic treatment, omadacycline treatment may provide advantages due to its PO as well as IV availability, and general well-tolerability.

## Data Availability

All data within this review are available within the cited references. No data were generated by the authors.
